# Impact of *CYP3A4* and *ABCB1* genetic variants on tacrolimus dosing in Greek kidney transplant recipients

**DOI:** 10.3389/fphar.2025.1538432

**Published:** 2025-03-19

**Authors:** Anna Tsironi, Konstantinos Lazaros, Effrosyni Mendrinou, Marios Papasotiriou, Stavroula Siamoglou, Kyriaki Kydonopoulou, Anne John, Alexandra Gerou, Spyridon Gerou, Bassam R. Ali, Aristidis G. Vrahatis, George P. Patrinos

**Affiliations:** ^1^ Laboratory of Pharmacogenomics and Individualized Therapy, Division of Pharmacology and Biosciences, Department of Pharmacy, School of Health Sciences, University of Patras, Patras, Greece; ^2^ Department of Informatics, Ionian University, Corfu, Greece; ^3^ Molecular Diagnostics Laboratory, INRASTES, National Centre for Scientific Research “Demokritos”, Athens, Greece; ^4^ Department of Nephrology and Kidney Transplantation, University Hospital of Patras, Patras, Greece; ^5^ ANALYSI Biomedical Laboratories S.A., Thessaloniki, Greece; ^6^ Department of Genetics and Genomics, College of Medicine and Health Sciences, United Arab Emirates University, Al-Ain, United Arab Emirates; ^7^ ASPIRE Abu Dhabi Precision Medicine Research Institute, Al-Ain, United Arab Emirates; ^8^ Zayed Center for Health Sciences, United Arab Emirates University, Al-Ain, United Arab Emirates; ^9^ Clinical Bioinformatics Unit, Department of Pathology, Faculty of Medicine and Health Sciences, Erasmus University Medical Center, Rotterdam, Netherlands

**Keywords:** kidney transplantation, tacrolimus, FK506, C/D ratio, Greek population

## Abstract

**Background:**

Tacrolimus, an approved first-line calcineurin inhibitor, is widely prescribed in organ transplantation to prevent allograft rejection. Its narrow therapeutic index requires precise management to achieve optimal dosing and to minimize adverse drug events (ADEs) while ensuring its therapeutic efficacy. Among several factors, genetic differences contribute significantly to the inter-individual and inter-ethnic variability in pharmacokinetics (PK) of tacrolimus in kidney transplant recipients. As a result, investigating the role of genetic variation in Greek transplant recipients becomes crucial to optimizing therapeutic strategies and enhancing the efficacy of immunosuppressive treatment.

**Hypothesis:**

Genetic variants which are known to influence the activity of enzymes or drug-transporters critical to tacrolimus pharmacokinetics, may significantly affect the required kidney post-transplant tacrolimus daily dose.

**Aim:**

To assess the correlation of *ABCB1* genetic variants (rs1128503, rs2229109) and *CYP3A4* (rs2242480, rs4986910) with tacrolimus dose-adjusted trough concentration (C_0_/D), in Greek kidney transplant recipients.

**Methods:**

Ninety-four unrelated Greek kidney transplant recipients were included in this study from the Department of Nephrology and Kidney Transplantation of the University General Hospital of Patras. Patients’ dose-adjusted trough levels were measured at five distinct time points after transplantation and analyzed in relation to the possible influence of *CYP3A4* and correlated with the abovementioned *ABCB1* genetic variants using standard genotyping analysis and Sanger sequencing.

**Results:**

The genetic variants rs1128503, rs2229109, rs2242480, rs4986910 did not show any significant association with the daily dosing requirements of tacrolimus for at least 1 year, in Greek patients who have undergone kidney transplant.

**Conclusion:**

It remains uncertain whether these genetic variants influence the assessment of the appropriate tacrolimus dosing 1 year after transplantation in Greek kidney transplant recipients.

## Introduction

Chronic kidney disease (CKD) represents a major global public health challenge, encompassing a diverse array of heterogeneous kidney disorders (Piras et al., 2022). It is a progressive chronic disease, characterized by abnormalities in kidney function and/or structure, for at least 3 months (Lameire et al., 2021). In the absence of effective intervention, CKD progresses to end-stage kidney disease (ESKD), necessitating life-sustaining treatments such as dialysis or kidney transplantation (Degraeve et al., 2020). Kidney transplantation is considered the preferred option, as it significantly extends patient survival and enhances quality of life (Ebid et al., 2022). Post-transplant management requires lifelong immunosuppressive therapy (IS) to avoid hyperacute and acute graft rejection, as well as, to sustain long-term graft function. The standard maintenance IS regimen typically consists of tacrolimus (TAC), a calcineurin inhibitor (CNI) that prevents T-cell activation and reduces the risk of organ rejection. This is combined with an anti-metabolite such as mycophenolate mofetil (MMF), and glucocorticoids (Hart et al., 2020). In most transplant centers, the standard initial dose of TAC is 0.15-0.2 mg/kg of body weight, administered in two separate doses.

Tacrolimus is characterized by a narrow therapeutic index and considerable intra- and inter-individual pharmacokinetic variability, emphasizing the critical need for therapeutic drug monitoring (TDM). Maintaining patients’ tacrolimus blood levels within the therapeutic range, is essential to minimize the adverse effects while optimizing therapeutic efficacy (Woillard et al., 2011). High intra-patient variability in tacrolimus exposure is recognized as a predictive marker for poor clinical outcomes (Mendoza Rojas et al., 2019). Tacrolimus pharmacokinetic variability is influenced by several factors, including its poor and highly variable oral bioavailability, food and drug-drug interactions, epigenetic modifications in metabolizing enzymes, genetic variants, demographic factors, gastrointestinal conditions, low serum protein and hematocrit levels, time post-transplantation, treatment non-adherence, and microbiota composition (Degraeve et al., 2020). These factors contribute to the complexity and interindividual variability in tacrolimus pharmacokinetics.

The pharmacokinetic profile of tacrolimus is affected by single nucleotide polymorphisms (SNPs) in genes encoding drug-metabolizing enzymes, drug transporters, drug receptors, targets. Tacrolimus is metabolized by gastrointestinal and hepatic CYP3A isoenzymes, mainly CYP3A5 and CYP3A4. The CYP3A5*3 allele is among the most extensively studied, with evidence supporting the use of *CYP3A5* genotyping as a preemptive strategy to guide tacrolimus dosing. CYP3A5 expressers who are carriers of at least one wild-type allele *1 generally require a starting dose 1.5 to 2 times higher than non-expressers (homozygous for the *3 allele) to achieve therapeutic blood concentrations (Birdwell et al., 2015). The CYP3A4*22 allele has also been shown to affect tacrolimus pharmacokinetics, though to a lesser extent. Carriers of this allele have decreased CYP3A4 enzyme activity, potentially resulting in elevated plasma levels of tacrolimus. Furthermore, tacrolimus is a substrate for P-glycoprotein, a multidrug efflux transporter encoded by the *ABCB1* gene. In kidney transplant recipients, the combination of recipient *CYP3A5* genotype and donor *ABCB1* genotype has been associated with an increased risk of tacrolimus-induced nephrotoxicity (Moore et al., 2012).

Although studies suggest that genetic variations in *CYP3A4* and *ABCB1* may influence tacrolimus plasma concentrations, the evidence remains inconsistent. For instance, certain variants in the *CYP3A4* gene, have been associated with altered enzyme activity and plasma drug levels, while other studies fail to confirm these associations (Liu et al., 2017a; Oetting et al., 2018a). Similarly, variations in the *ABCB1* gene, have been linked to differences in drug absorption and distribution, but these findings have not been consistently observed across studies (Staatz et al., 2010).

Currently, limited information is available regarding Greek kidney transplant recipients. Therefore, in this study, we further evaluate the potential association between the genetic variants located in the *ABCB1* gene, namely, rs1128503 (g.87550285A>G), rs2229109 (g.87550493C>T) and *CYP3A4* gene, namely, rs2242480 (g.99763843C>T), rs4986910 (g.99760901A>G), based on GRCh38 reference genome, and the daily tacrolimus dose requirements in this population, during the first 12 months following kidney transplantation.

## Materials and methods

### Study population

A retrospective study was conducted in 94 unrelated Greek adult kidney recipients treated at the Department of Nephrology and Kidney Transplantation of the University General Hospital of Patras. Each participant had a comprehensive medical history, received a renal transplant from either living or deceased donors, and was maintained on tacrolimus-based immunosuppressive therapy for at least 1-year post-transplant. The initial pre- and post-operative dose was administered *per os* based on body weight, ranging from 0.15 to 0.2 mg/kg/day for Prograf^®^. Patients were monitored weekly during the first month post-transplant, biweekly until 3 months, and every 2–3 months thereafter. Therapeutic drug monitoring (TDM) was performed with the ARCHITECT Tacrolimus Assay, which is based on chemiluminescent microparticle immunoassay (CMIA) technology, to adjust patients’ doses within the therapeutic range, at five specific time points (first time, after 1 month, 3 months, 6 months, and finally, 1-year post-transplant). Blood samples were collected for genotyping analysis during routine follow-up visits. Medical records were reviewed to collect patient characteristics, TAC doses (D) and trough blood levels (C_0_). Dose-adjusted concentrations (C_0_/D) were also estimated across these time points.

All participants provided written informed and signed consent, and the study received approval from the Research Ethics Committee of the University of Patras, Rion, Greece (17/03/2017). All the experiments involving human subjects were conducted in compliance with the principles outlined in the Declaration of Helsinki.

### Genotyping analysis

Genomic DNA from recipients was extracted from peripheral blood leukocytes using a standard phenol/chloroform protocol, followed by the assessment of DNA purity and concentration. PCR amplification conditions are available upon request.

The *ABCB1* variants rs1128503 and rs2229109, were genotyped by PCR-RFLP using the *EcoO109I* and *AcuI* restriction endonucleases (NEW ENGLAND BioLabs^®^ Inc.) respectively. For *CYP3A4* variants rs4986910 and rs2242480, a PCR-based conventional Sanger sequencing method was utilized with the Big Dye Terminator v3.1 Cycle Sequencing Kit (Applied Biosystems, CA, USA). Capillary electrophoresis was performed on the ABI Prism 3130xl DNA Analyzer (Applied Biosystems).

### Computational and statistical analysis

The allelic and genotype frequencies were estimated using the gene counting method. Hardy–Weinberg equilibrium was determined using the chi-square goodness of fit test (1 degree of freedom). Continuous variables are presented as means with standard deviations, and categorical data are reported as frequencies and percentages.

Computational analysis was conducted using data science and machine learning libraries in Python, with data preprocessing managed through the pandas library (McKinney, 2011). For three of the four genetic variants—rs2229109, rs1128503, and rs2242480—the preprocessing focused on selecting columns associated with the C_0_/D ratio at specific time points and the corresponding genotypes for each variant. Samples with unknown or highly unique genotypes, which could introduce class imbalance, were excluded to maintain data integrity. As a result, three distinct datasets were generated, each representing one of the variants. These datasets contain six columns: one identifying the genotype or sample tag, and five tracking the C_0_/D ratio at key time intervals (first time, 1 month, 3 months, 6 months, and 1 year). The dataset corresponding to rs2242480 comprises of 93 patient samples, while rs1128503 and rs2229109 variants were analyzed in 94 patient samples. The target variable for rs2242480 and rs2229109 is binary, reflecting the presence of two distinct genotypes. In contrast, the target variable for rs1128503 is multi-class, as it represents three unique genotypes. Classic machine learning and statistical analysis methods are applied to each dataset to demonstrate that no significant relationship exists between the input features (C_0_/D) and the genotype of the respective samples.

To evaluate the association between samples and their corresponding genotypes, dimensionality reduction was applied to each of the three datasets using Principal Component Analysis (PCA). Data visualization was conducted utilizing the matplotlib and seaborn libraries (Tosi, 2009; Waskom, 2021). PCA was employed to reduce the dimensionality of the data, and two-dimensional visualizations were generated for each dataset to facilitate a thorough analysis (Greenacre et al., 2022).

To further assess the association and predictive power of the five retained features in each dataset with respect to the target variable, the PyCaret Python package was utilized (Ahmad and Fahmi, 2024). For the datasets corresponding to rs2242480 and rs2229109, binary classifiers were employed, while for rs1128503, multi-class classifiers were applied. This approach facilitated the evaluation of multiple machine learning models, systematically comparing their performance to determine their ability to predict outcomes based on the selected features. To further investigate the relationship between samples and their corresponding genotypes, Pearson correlation heatmaps were generated for each dataset. This analysis was performed for all three datasets to explore potential correlations between the input features (C0/D) and the target variable, the genotype. By visualizing the correlation matrices as heatmaps, any patterns or associations present within the data could be examined in greater detail. This approach enabled a systematic assessment of the strength and nature of the correlations, providing further evidence to support the hypothesis that no significant relationship exists between the features and the genotypes in each dataset.

## Results

### Patient demographic characteristics

The characteristics of the 94 Greek kidney transplant recipients included in this study are summarized in Table 1. Of the patients, 61 (64.9%) were male and 33 (35.1%) were female, with a mean age of 41.87 ± 14.01 years at the time of their first kidney transplant. The kidney transplant was received from both living related 19 (20.2%) and deceased donors 75 (79.8%). Diagnoses of kidney-related conditions leading to ESKD were established based on clinical history, physical examination, and endoscopic and histological evaluations. All patients subsequently underwent kidney transplant and received life-long post-transplant tacrolimus therapy.

**TABLE 1:** Patient characteristics (n = 94).

Characteristic	Value
All	94
Male	61 (64.9%)
Female	33 (35.1%)
Onset at the 1st transplantation y (SD)	41.87 ± 14.01
*Transplantation (%)*	
First	75 (79.8%)
Second	18 (19.1%)
Third or more	1 (1.1%)
*Donor type n (%)*	
Living related	16 (17%)
Living unrelated	3 (3.2%)
Deceased	75 (79.8%)
*Family history n (%)* ^*a*^	
Yes	18 (19.1%)
No	76 (80.9%)

Values are n (%) unless otherwise defined.

^a^ Family history refers to cases where genetic factors contributed to renal failure and the need for transplantation.

### Allelic and genotype frequencies in kidney transplant recipients

The genotype and allele frequencies of the four genetic variants among these 94 Greek patients are presented in Table 2. All genotype frequencies adhered to Hardy-Weinberg equilibrium (p-value>0.05) except for the rs1128503 variant (χ^2^ > 3.841). Allele frequencies for most variants, with the exception of rs2229109, were consistent with those previously reported for Caucasian populations in public databases such as Ensembl. For the rs1128503 variant, the genotype distribution was as follows: 17 (18.09%) patients had the G/G genotype, 60 (63.83%) were heterozygous (A/G), and 17 (18.09%) had the A/A genotype. The total allele frequencies were 50% for the G allele and 50% for the A allele. Regarding the rs2229109 variant, 74 (78.72%) had the wild-type C/C genotype, while 20 (21.28%) were heterozygous (C/T). The total allele frequencies were 89.36% for the C allele and 10.64% for the T allele. For the rs4986910 variant, all patients had the wild-type A/A genotype, resulting in an allele frequency of 100% for the A allele. Lastly, for the rs2242480 variant, 75 (79.79%) had the C/C genotype, 18 (19.15%) were heterozygous (C/T), and 1 (1.06%) had the T/T genotype. The total allele frequencies were 89.36% for the C allele and 10.64% for the T allele.

**TABLE 2:** Genotype allele frequency, and Hardy-Weinberg equilibrium (HWE) assessment of the four genetic variants.

Genetic variant	Gene	Allele frequency		p-value	Genotype results (%)	HWE (degrees of freedom:1) (χ^2^)
		Caucasian population* (reference)	Greek kidney transplant recipients			
rs1128503	*ABCB1*	G: 57.1%	G: 50%	0.3950	G/G:17 (**18.09**)	**>3.841**
		A: 42.9%	A: 50%		A/G: 60 (**63.83**)	
					A/A: 17 (**18.09**)	
rs2229109	*ABCB1*	C: 98.5%	C: 89.36%	**0.0050**	C/C:74 (**78.72**)	1.3322
		T: 1.5%	T: 10.64%		C/T: 20 (**21.28**)	
					T/T: 0 (**0**)	
rs2242480	*CYP3A4*	C: 94.4%	C: 89.36%	0.3106	C/C: 75 **(79.79%)**	0.0048
		T: 5.6%	T: 10.64%		C/T: 18 (**19.15**)	
					T/T: 1 (**1.06**)	
rs4986910	*CYP3A4*	A: 98.5%	A: 100%	1.0000	Α/Α: 94 (**100**)	
		G: 1.5%	G: 0%		Α/G: 0 (**0**)	
					G/G: 0 (**0**)	

*Allele frequencies are provided from Ensembl database, p-value shows the statistical comparison of allele frequency.

### Association of the genetic variants with the Co/D ratio of tacrolimus in renal transplant recipients

At the first time, and months 1, 3, 6 and 12 after kidney transplantation, the average tacrolimus doses (mg/day) were 6.39 ± 3.26, 6.14 ± 3.58, 5.51 ± 3.23, 4.82 ± 2.95, and 4.26 ± 2.68, respectively. The tacrolimus trough concentrations (C_0_) and dose-adjusted trough concentration ratios (C_0_/D) are shown in Table 3.

**TABLE 3:** Tacrolimus dose (mg/day), trough concentration (ng/mL) and Co/D ratio (ng/ml per mg/day) in Greek kidney transplant patients at the first time, months 1, 3, 6 and 12 after transplantation.

Tacrolimus Dose, Trough Concentration, and C0/D Ratio (First time, months 1, 3, 6, 12)	Mean (SD)
Initial Tac D mg/day mean (SD)	6.39 ± 3.26
1-month Tac D, mg/day mean (SD)	6.14 ± 3.58
3-months Tac D mg/day mean (SD)	5.51 ± 3.23
6-months Tac D mg/day mean (SD)	4.82 ± 2.95
1-year Tac D mg/day mean (SD)	4.26 ± 2.68
Initial Tac C_0_ ng/mL mean (SD)	8.29 ± 4.37
1-month Tac C_0_ ng/mL mean (SD)	7.75 ± 3.08
3-months Tac C_0_ ng/mL mean (SD)	7.12 ± 2.60
6-months Tac C_0_ ng/mL mean (SD)	6.75 ± 2.61
1-year Tac C_0_ ng/mL mean (SD)	6.03 ± 2.35
Initial C_0_/D ratio ng/mL per mg/day mean (SD)	1.61 ± 1.08
1-month C_0_/D ratio ng/mL per mg/day mean (SD)	1.67 ± 1.11
3-months C_0_/D ratio ng/mL per mg/day mean (SD)	1.66 ± 0.90
6-months C_0_/D ratio ng/mL per mg/day mean (SD)	1.97 ± 1.37
1-year C_0_/D ratio ng/mL per mg/day mean (SD)	1.95 ± 1.31

Abbreviations: D, daily dose of tacrolimus; C_0_, blood trough concentration of tacrolimus; SD, standard deviation.

**FIGURE 1:**
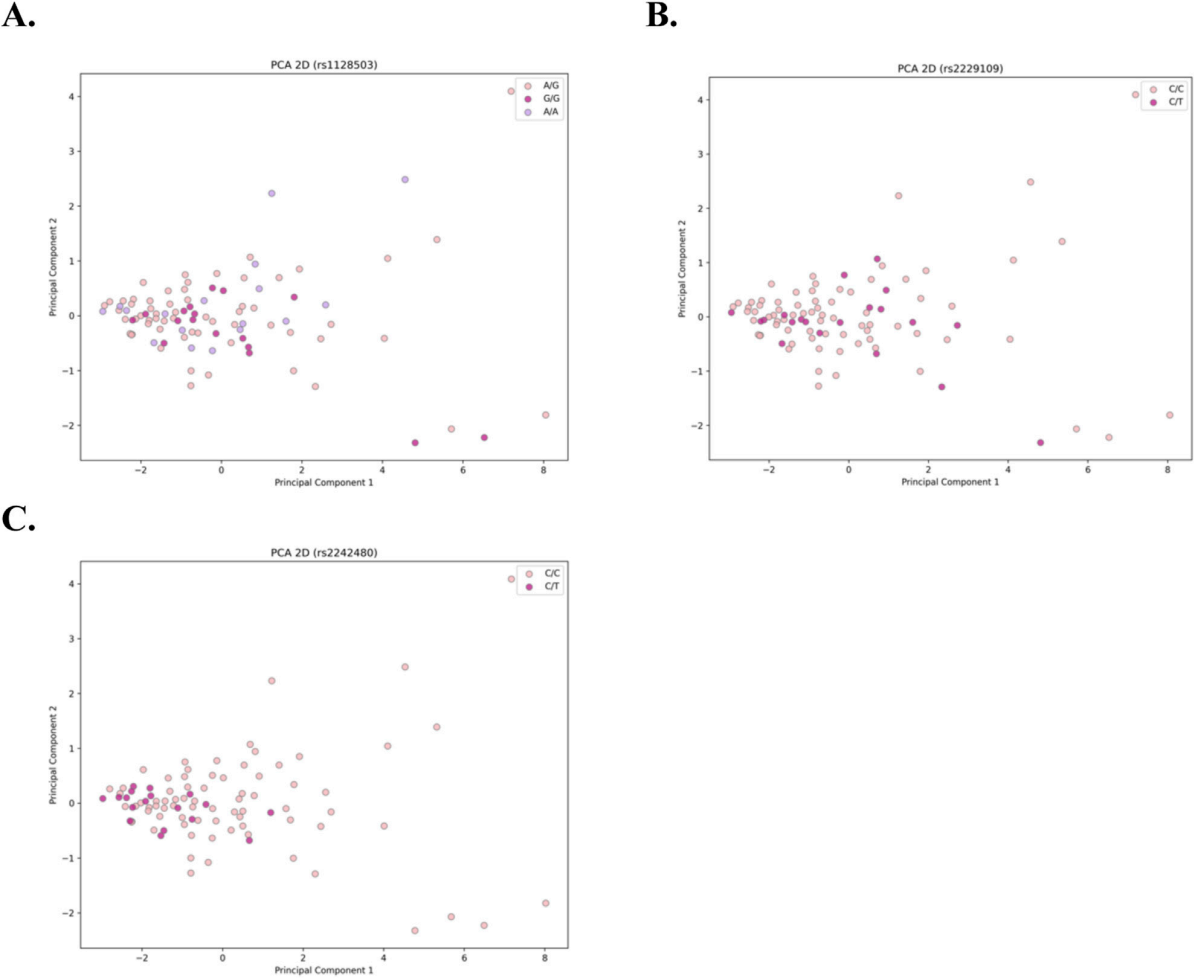
Two-dimensional PCA plots for the 3 genetic variants: **(A)** rs1128503, **(B)** rs2229109, and **(C)** rs2242480. Each dot represents an individual sample, with colors denoting the corresponding genotype. The absence of distinct clustering or separation among samples across all cases indicates no significant association between the C0/D ratio and genotype.

The results of the dimensionality reduction algorithms are presented in Figure 1 for the three genetic variants **(A)** rs1128503, **(B)** rs2229109, and **(C)** rs2242480. In all plots, each dot represents a sample, with colors corresponding to the genotype of each sample. Across all cases, there is no clear pattern of separation between samples, which serves as a clear indication that the C_0_/D ratio does not exhibit a significant association with genotype.

**FIGURE 2:**
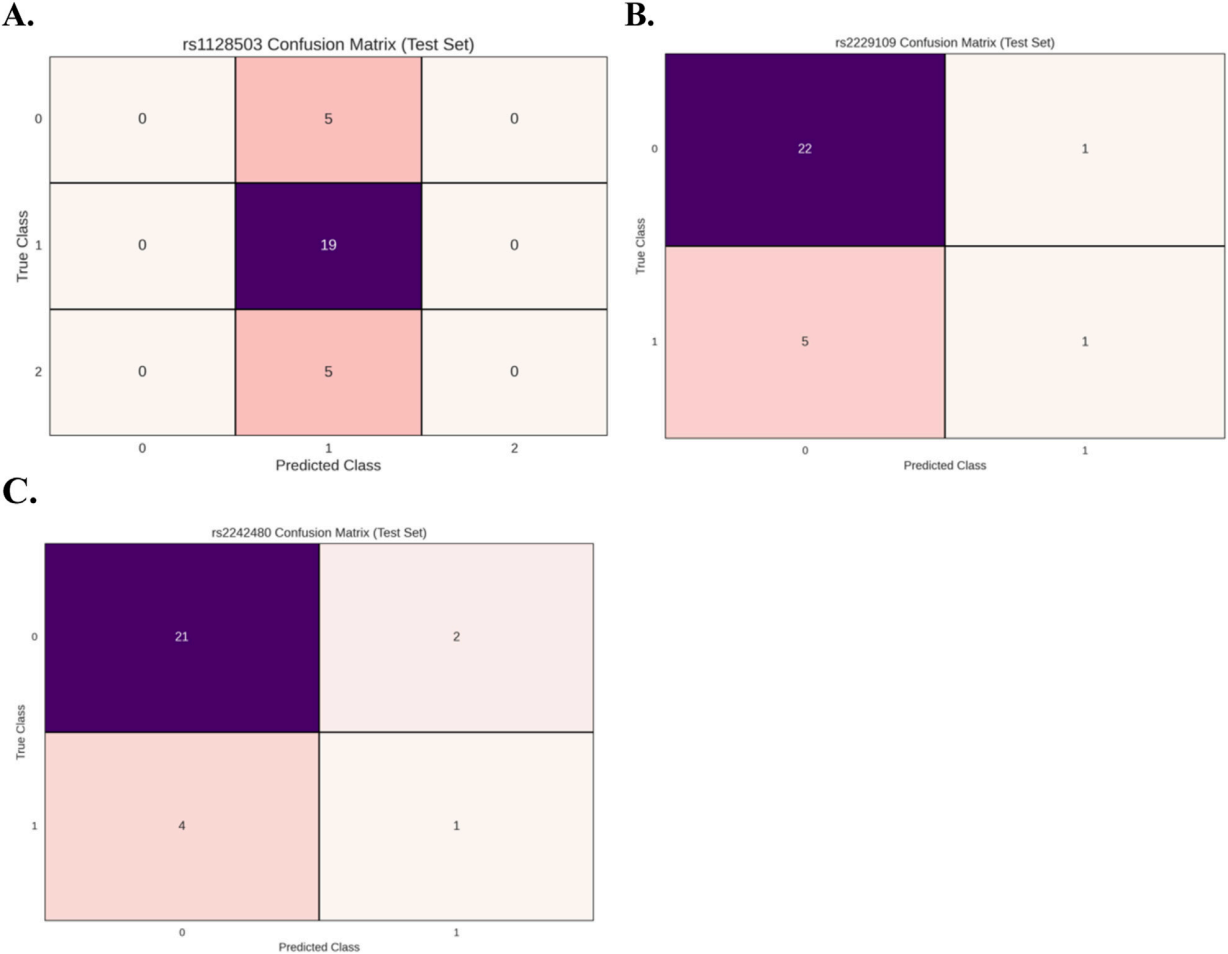
Confusion matrices for the best-performing models evaluated using PyCaret for the 3 genetic variants: **(A)** rs1128503 (multi-class classification), **(B)** rs2229109 (binary classification), and **(C)** rs2242480 (binary classification). The results indicate suboptimal model performance across all datasets. In binary classification cases, the models exhibit a bias toward class 0, while in the multi-class case, the model demonstrates a bias toward class 1. These findings highlight the lack of predictive power of features derived from the C0/D ratio in determining genotype.

Figure 2 presents the results of the PyCaret model evaluations, showing the confusion matrices for the best-performing model of the three genetic variants **(A)** rs1128503, **(B)** rs2229109, and **(C)** rs2242480. Across all three datasets, the confusion matrices suggest that the models underperform, failing to achieve optimal results for binary and/or multiclass classification. In both binary classification instances, the classifiers are biased towards class 0 whereas in the multiclass instance the model is biased towards class 1. These patterns further indicate that the features derived from the C_0_/D ratio do not significantly contribute to predicting the genotype in any of the cases.

**FIGURE 3:**
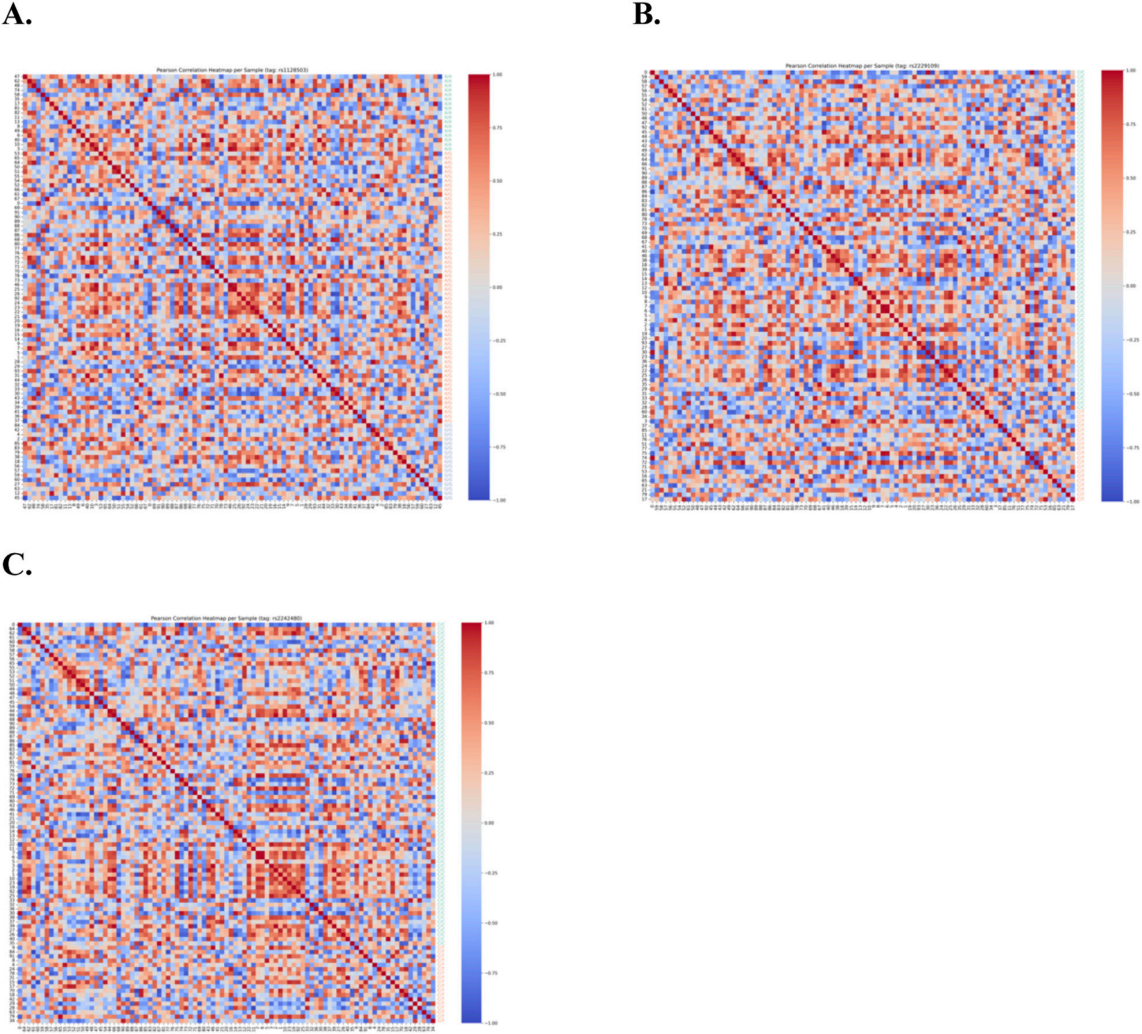
Per-sample Pearson correlation heatmaps for the three datasets: **(A)** rs1128503, **(B)** rs2229109, and **(C)** rs2242480. The heatmaps reveal no distinct correlation patterns between samples and their genotypes. While some samples display strong positive pairwise correlations, others exhibit weak or negligible correlations, regardless of genotype. This inconsistency further supports the conclusion that the C0/D ratios are not significantly associated with genotype across the datasets.

Figure 3 presents the per-sample correlation heatmaps for the three datasets **(A)** rs1128503, **(B)** rs2229109, and **(C)** rs2242480 under computational analysis. No distinct correlation pattern is observed between samples in relation to their genotypes. Some samples exhibit strong positive pairwise correlations, while others show weak or no correlation, regardless of whether they share the same genotype. This lack of a consistent correlation pattern further suggests that genotype does not have a significant association with the C_0_/D ratios across the different time points.

## Discussion

The clinical use of tacrolimus is challenging due to its narrow therapeutic range and interindividual pharmacokinetic variability. While therapeutic drug monitoring is commonly utilized to optimize dosing, the risk of underimmunosuppression and acute rejection remain significant concerns. Notably, tacrolimus trough levels can exhibit considerable variability, even during routine monitoring in the stable post-transplant phase (Mohamed et al., 2019). The role of P-glycoprotein and CYP3A enzymes in the pharmacokinetics of tacrolimus has long been acknowledged, as they are key determinants of its metabolism and transport (Hesselink D. et al., 2003). Interestingly though, the current retrospective study failed to demonstrate significant association between genetic variants in the *ABCB1* and *CYP3A4* genes and the pharmacokinetic profile of tacrolimus in Greek renal transplant recipients, a finding that is consistent with previous cohort studies in other populations (Table 4). Also, the number of cases collected within the scope of the present study (n = 94) is sufficient to suggest that these specific variants are not really clinically relevant in the Greek population.

**TABLE 4:** Summary of studies investigating the association between *ABCB1* and *CYP3A4* variants and tacrolimus pharmacokinetics in kidney transplant recipients.

Ethnicity	No. of patients	Organ transplanted	Phenotype Category	Tacrolimus PK Correlation (Yes/No)	Association (p-value)	Allele frequency	References
*ABCB1*							
rs1128503							
European	91	Kidney	Dosage	Yes	<0.05	A = 0.32	Kravljaca et al. (2016b)
Latino	108	Kidney	Metabolism/PK	Yes	0.047	A = 0.4	Cusinato et al. (2014)
Multiple groups, Malay, Chinese, Indian	45	Kidney	Metabolism/PK	No	0.2	A = 0.611	Hamzah et al. (2014)
East Asian	216	Liver	Metabolism/PK	No	>0.05	G = 0.352	Shi et al. (2013)
East Asian	249	Kidney	Metabolism/PK	No	>0.05	G = 0.408	Ro et al. (2012)
East Asian	155	Kidney	Metabolism/PK	No	>0.05		Li et al. (2015b)
European	98	Liver	Dosage	No	N.S.		Gómez‐Bravo et al. (2013b)
East Asian	276	Kidney	Metabolism/PK	No	>0.05	A = 0.435	Liu et al. (2017b)
European	91	Kidney	Dosage	No	>0.05	A = 0.32	Kravljaca et al. (2016c)
East Asian	53	Liver	Dosage	No	<0.0187	G = 0.406	Zhang et al. (2011a)
East Asian	132	Kidney	Dosage	No	<0.001	A = 0.582	Kim et al. (2012)
East Asian	80	Kidney	Metabolism/PK	No	<0.001	A = 0.613	Han et al. (2013)
rs2229109							
European	369 (Replication cohort) 330 (Discovery cohort)	Kidney	Efficacy	Yes	0.0142 and 0.0288	T = 0.06 and T = 0.036	Woillard et al. (2018a)
Multiple groups	165	Kidney	Efficacy	No	0.1 and 0.3		Hu et al. (2019)
*CYP3A4*							
rs2242480							
East Asian	240	Kidney	Metabolism/PK	No	>0.05	T = 0.27	(C.-J. Li et al., 2014)
European	1,560	Kidney	Metabolism/PK	Yes	6.08E-98	C = 0.068	Oetting et al. (2018b)
rs4986910							
Allele Frequency: 0%–1.8%; rare variant with limited data; Tacrolimus PK Correlation: Limited evidence due to low allele frequency (Saiz-Rodríguez et al., 2020)							
Multiple groups	64	Kidney	Dosage, Metabolism/PK	Yes	N.A.		Hesselink (2003a)

Tacrolimus undergoes counter-transport by P-glycoprotein, and the influence of *ABCB1* genetic variants on P-gp expression, function, and its plasma concentration remains controversial. The influence of *ABCB1* genetic variants in determining tacrolimus dosing has been questioned in previous studies, with contradictory findings. While studies have reported associations, others have found no significant link between *ABCB1* polymorphisms and tacrolimus dose requirements (Haufroid et al., 2006; Mourad et al., 2005). Consistent with these findings, our study observed no statistically significant association between *ABCB1* genotypes and any tacrolimus pharmacokinetic variables. One possible explanation is that the effects of *ABCB1* polymorphisms may be masked by the influence of more pronounced CYP3A enzyme polymorphisms (Zhang et al., 2011a).

The variability in the relationship between *ABCB1* variants and tacrolimus response, may also be influenced by differences in immunosuppression protocols across studies, ethnic-related genetic diversity, or limitations due to small sample sizes (Gómez‐Bravo et al., 2013a). For instance, the variant A allele frequency for rs1128503 ranges from 10% to 63% across populations, with a frequency of 43% in Caucasians and 14% in Africans (Ensembl). In contrast, our cohort shows an 18.09% frequency of the A/A homozygous genotype for rs1128503, compared to 1.09% reported by Kravljaca et al. (2016a). This deviation suggests potential selective pressures specific to our cohort of Greek transplant patients, as indicated by Hardy–Weinberg disequilibrium for rs1128503.

The impact of rs1128503 variant on tacrolimus concentrations remains uncertain. While some studies have reported a relatively low association between this SNP and elevated tacrolimus blood concentrations (Cheung et al., 2006; Fredericks et al., 2006a), others have found no significant effect (Jun et al., 2009; Li et al., 2015a). In our study, we observed a negative association between rs1128503 variant and tacrolimus C_0_/D ratios across five distinct time points. Interestingly, findings from Zhang et al. (2011b) indicated that individuals with the A/A genotype, had higher tacrolimus blood levels compared to those with the G/G genotype.

The complex role of *ABCB1* variants in tacrolimus pharmacokinetics is further explored through haplotype studies of the rs1045642, rs2032582, and rs1128503 variants. In a cohort of 81 kidney transplant recipients, the C-G-C haplotype was found to be associated with higher tacrolimus dose requirements compared to the T-T-T haplotype (Anglicheau et al., 2003). These SNPs are in varying degrees of linkage disequilibrium (LD), raising the question whether tacrolimus pharmacokinetics is influenced by a single variant or the combined haplotype (Grinyó et al., 2008; Wang et al., 2005). rs2032582 and rs1045642 have shown strong LD, with reported *r* ^2^ or D′ values greater than 0.8 (Bochud et al., 2008; Grinyó et al., 2008). While rs2032582 is a nonsynonymous SNP, rs1045642 and rs1128503 are synonymous, suggesting that rs2032582 may have a more direct functional impact on ABCB1 activity. Despite these associations, only rs1045642 has been identified as influencing *ABCB1* gene expression (Hoffmeyer et al., 2000). A study with 206 renal transplant recipients found no significant association with *ABCB1* haplotypes, especially after adjusting for *CYP3A5* status (Fredericks et al., 2006b). Thus, it remains unclear whether these effects are driven by a single SNP or the combined haplotype, as other genetic and clinical factors may also contribute to interindividual variability in tacrolimus response.

The rs2229109 variant was detected in 10.64% of our cohort, a frequency that is markedly higher than the 1.5% reported in broader Caucasian populations. This could be attributed to the small sample size or the ethnically homogenous nature of the Greek cohort. The ABCB1 protein encoded by the wild type rs2229109 allele, is more efficient at transporting tacrolimus compared to the variant allele, suggesting that the p.Ser400Asn amino acid substitution may impair ABCB1’s ability to efflux tacrolimus. This alteration may partially account for interindividual differences in drug response (Dessilly et al., 2014). Additionally, other transporters from the ABC or SLC families, such as MRP2, MRP4, and MATE, which are expressed in the kidney, could minimally contribute to tacrolimus transport (Woillard et al., 2018a). Research on the impact of rs2229109 genetic variant on tacrolimus response is limited. Hu and coworkers (2018) reported that the C/T genotype of rs2229109 did not increase the risk of transplant rejection compared to the C/C genotype, further highlighting the need for more comprehensive studies.

In our study, the rs4986910 variant was not detected among Greek transplant recipients, despite its 1.5% frequency in Caucasians. It can alter the enzyme structure, potentially affecting function, though further investigation is needed to confirm this. According to a study by Hesselink D. A. et al. (2003), organ transplant recipients who carry the variant allele, may require lower tacrolimus doses compared to homozygotes for the wild type allele. However, variant’s low prevalence, limits available clinical evidence on its effect on tacrolimus dosing.

Our findings align with previous evidence indicating no significant association between the C_0_/D ratio of tacrolimus and rs2242480 in Greek renal transplant patients. Specifically, Li and coworkers (2014) reported that the C/C wild-type genotype was not associated with a likelihood of achieving target concentrations of tacrolimus when compared to the C/T + T/T genotypes in kidney transplant recipients. Similarly, Liu and coworkers (2019) found no significant difference in tacrolimus concentrations between children with nephrotic range proteinuria carrying the T allele and those with the C/C genotype. These findings further support our observation that the rs2242480 variant, does not appear to play a key role in determining tacrolimus plasma levels. Larger prospective studies are necessary to further explore its impact on tacrolimus pharmacokinetics in transplant recipients. This conclusion is further substantiated through the application of well-established statistical and machine learning methodologies, which clearly demonstrated that the C_0_/D ratio lacks predictive power in relation to the target variable, the genotype of each patient.

The primary limitations of this study include its single-center retrospective study design and a relatively small cohort size. Additionally, the potential combined effects of *CYP3A4*, *CYP3A5*, and *ABCB1* genetic variants on tacrolimus pharmacokinetics were not investigated. Also, the determination of Greek ancestry was based on self-reported ethnicity, which could have been strengthened by genetic ancestry verification. Moreover, the potential influence of co-medications, particularly those that inhibit or induce CYP3A enzymes, on tacrolimus metabolism was not specifically analyzed, which may have contributed to variability in drug response. Given the population-specific genetic variability, genetic stratification is crucial for more accurate results. Future prospective, multi-center studies with a larger cohort of transplant recipients are necessary to develop a reliable tool for monitoring immune responses.

This study utilized a candidate-gene approach, a hypothesis-driven methodology commonly used to investigate associations between specific single nucleotide variants and complex outcomes. While this approach is valuable, it limits the potential for discovering other relevant pharmacogenes, or rare variants that may be associated with clinical outcomes. To address these limitations, future research would benefit from techniques such as whole genome sequencing, which provide a more agnostic and comprehensive genetic insight. However, these approaches require large patient cohorts to detect statistically significant associations, given the low frequency and penetrance of such genetic variations (Woillard et al., 2018b).

## Conclusion

The present study did not find a significant association between *ABCB1* rs1128503, rs2229109 and *CYP3A4* rs2242480, rs4986910 variants and tacrolimus exposure in Greek renal transplant patients. Based on the findings, no significant correlation between these genetic variants and tacrolimus dosing requirements was revealed. These results underscore the complexity of pharmacogenomic factors on tacrolimus metabolism and highlight the necessity for further research to uncover additional factors contributing to the variability in drug response among renal transplant recipients. Such insights are crucial for advancing personalized medicine, enabling tailored therapeutic strategies, and ultimately improving clinical outcomes in kidney transplantation.

## Data Availability

The raw data supporting the conclusions of this article will be made available by the authors, without undue reservation.
